# Supplementary material for: Design and Analysis Strategies for Pooling in High Throughput Screening: Application to the Search for a New Anti‐Microbial
Source: Stat Med. 2026 Oct 16;45(23-24):e70778. doi: 10.1002/sim.70778 (PMC13674217; doi:10.1002/sim.70778)
Supplement: Supplementary file 1 [file SIM-45-0-s001.pdf]

Design and Analysis Strategies for Pooling in High Throughput Screening: Application to the Search for a New Anti-Microbial - Supplementary Document

1 CRowS vs. Orthogonal Pooling

Here we report results of CRowS vs. Orthogonal Pooling (Figure 1, where CRowS is analyzed using the Gauss-Lasso with $\tau_\lambda = \max(\hat{\beta}_\lambda)$ while the Orthogonal Pooling design is analyzed using a traditional, non-statistical modeling method described below.

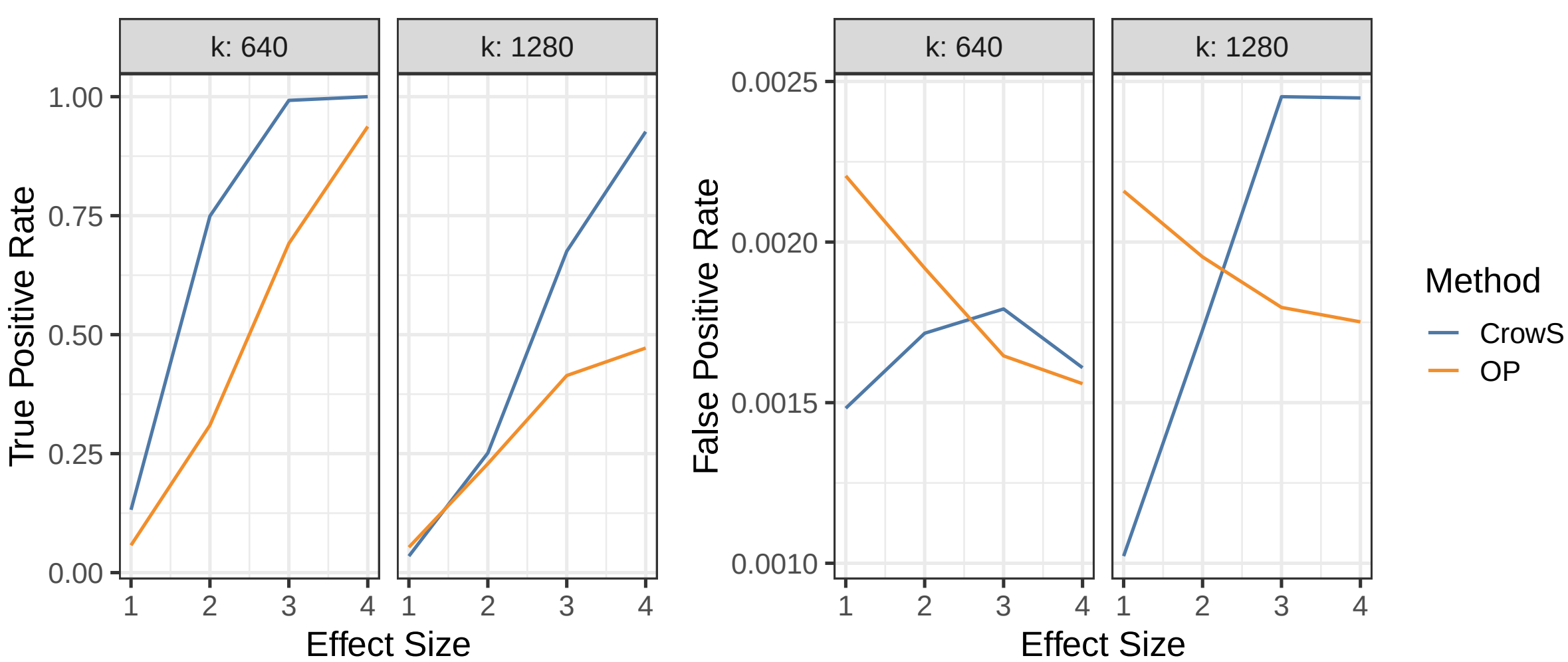

Figure 1: Comparison of FPR and TPR between CRowS and Orthogonal Pooling designs using the λ-specific Gauss-Lasso with $\tau_\lambda = \max(\hat{\beta}_\lambda)$ primary thresholding criterion for CRowS.

For orthogonal pooling designs, the data was generated in a manner identical to our primary simulations, with effect sizes $\beta = (1, 2, 3, 4)$ and error $\sigma^2 = 1$. The simulations

were carried out with 10,000 iterations, and, at each iteration, a compound was designated a hit if the values of both observed wells (y_i, y_j) in which the compound was present were greater than the 95th percentile $(y_{0.95})$ of all observed well values.

2 Proof-of-concept Screening Results for $(n = 320, k = 1280, c = 8)$

Here we provide a plot (Figure 2) of the results of the $(n = 320, k = 1280, c = 8)$ proof-of-concept screen. Though there is some visual evidence that the screen identified the pseudo-hit and true hit, we were unable to verify this using the Gauss-Lasso modeling.

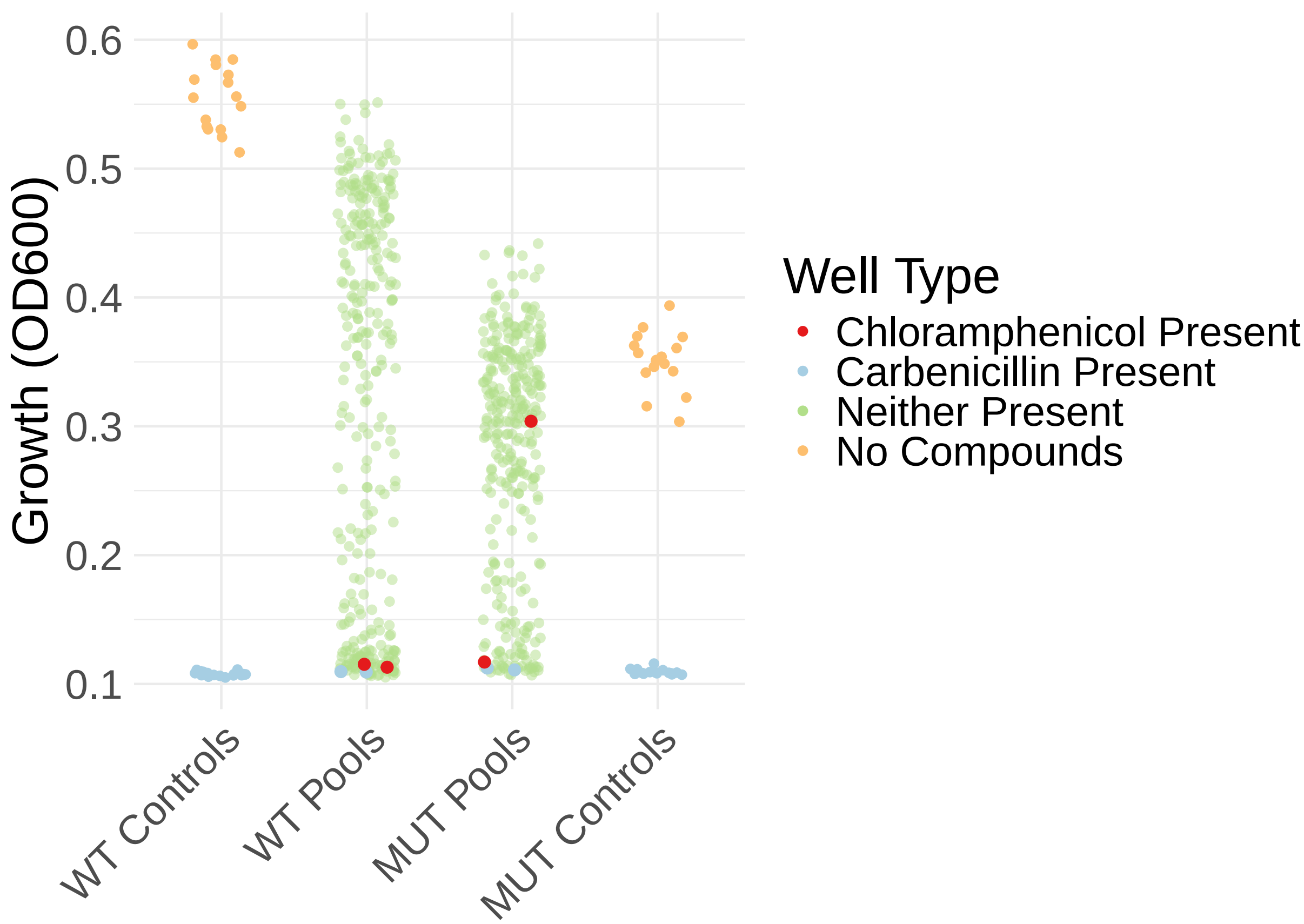

Figure 2: Representation of the amount of cell growth for 32 WT Controls, the 320 WT pooled wells, the 320 MUT pooled wells, and the 32 MUT Controls. The pooled wells studied 1,280 compounds. The wells are colored by whether they included the pseudo-hit (Carbenicillin) or the true hit (Chloramphenicol). For the Controls, the Carbenicillin wells included *only* Carbenicillin; for the pooled wells, each well included 8 different compounds.

3 Boxplots from Small Screen

In Figure 3 we provide boxplots from the small screen described in Section 5.2 in the paper.

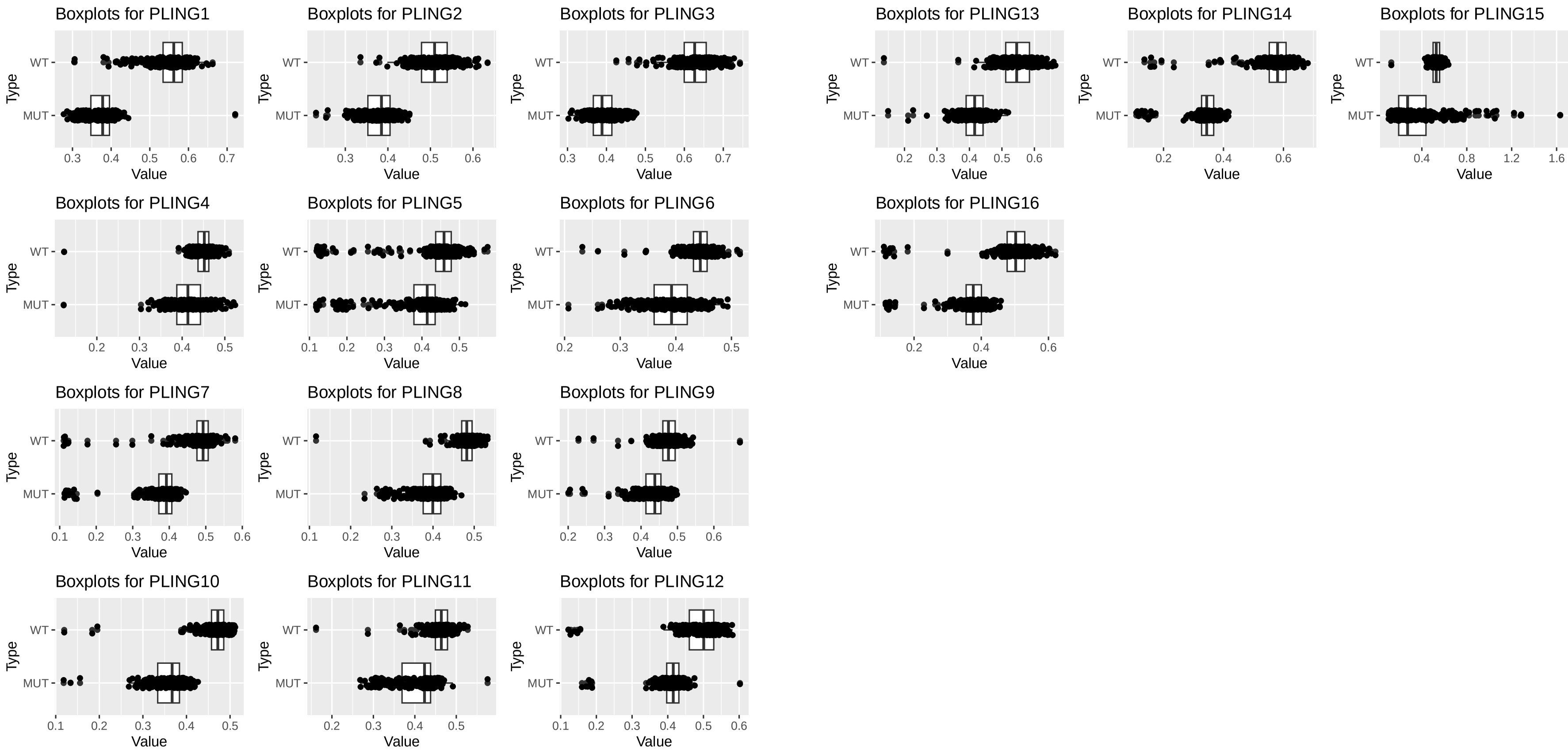

Figure 3: Raw data from the screen described in Section 5.2 of the document.

4 Interactions

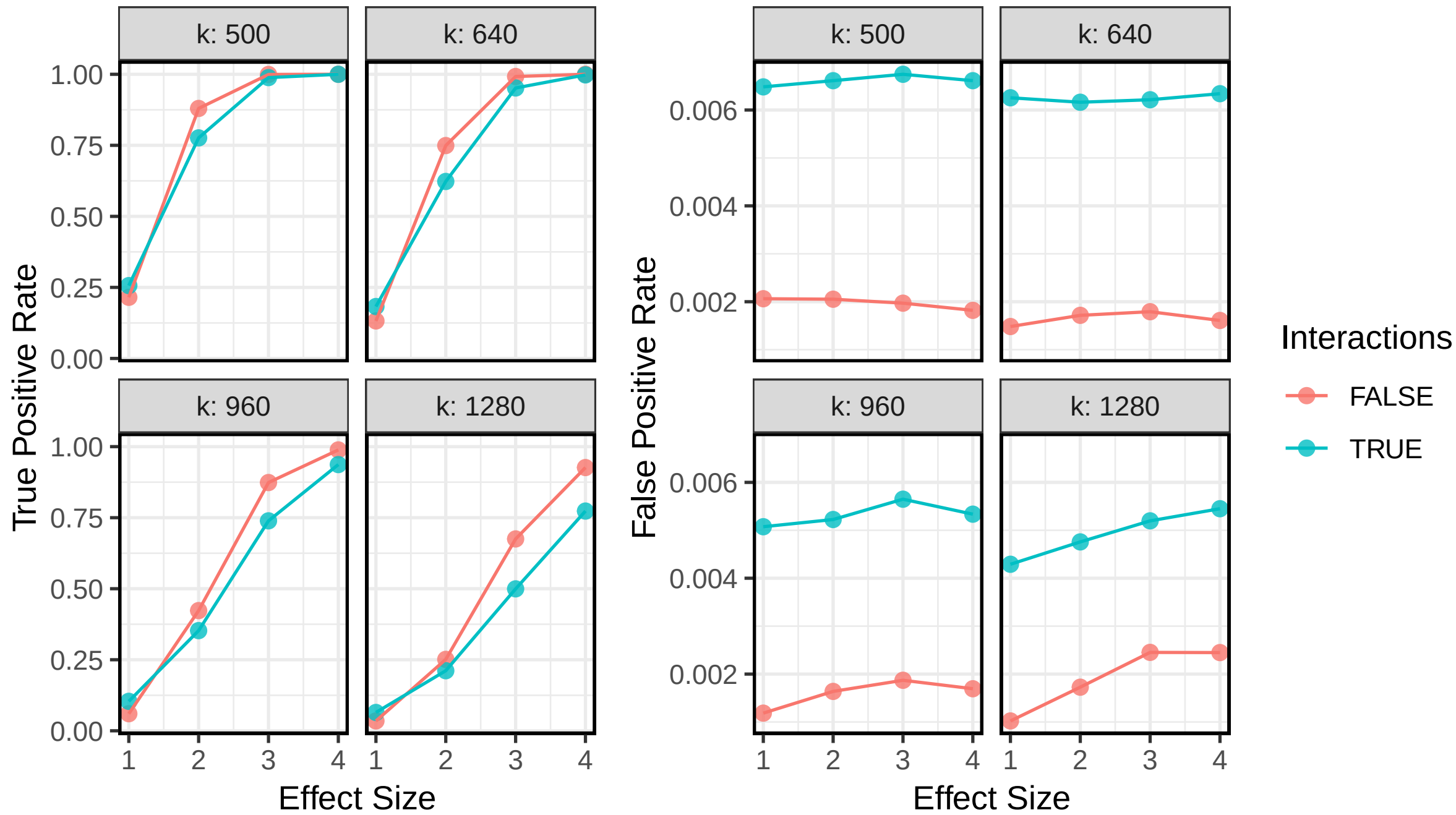

Figure 4: Comparison of FPR and TPR when compounds have the possibility of interacting with each other versus not, using CRowS designs the λ-specific Gauss-Lasso with $\tau_\lambda = \max(\hat{\beta}_\lambda)$ primary thresholding criterion. See Section 6 of the paper for details.
